# Contemporary management of TMJ involvement in JIA patients and its orofacial consequences

**DOI:** 10.1186/s13167-016-0061-7

**Published:** 2016-06-02

**Authors:** Priit Niibo, Chris Pruunsild, Ülle Voog-Oras, Tiit Nikopensius, Triin Jagomägi, Mare Saag

**Affiliations:** 1 Institute of Dentistry, University of Tartu, Raekoja plats 6, 51003 Tartu, Estonia; 2 Department of Pediatrics, University of Tartu, Institute of Clinical Medicine, N. Lunini 6, 51014 Tartu, Estonia; 3 Estonian Genome Center, University of Tartu, Riia 23b, 51010 Tartu, Estonia

**Keywords:** Biomarkers, Craniofacial growth, Diagnostics, Imaging methods, Juvenile idiopathic arthritis, Oral health, Prevention, Temporomandibular joint, Treatment

## Abstract

Juvenile idiopathic arthritis is the most common chronic rheumatic condition during childhood. Temporomandibular joint arthritis is frequently asymptomatic. When it takes place during childhood, it may affect condylar growth; therefore, these children are at risk of unfavorable long-term outcomes from the associated joint damage. The etiology is not completely understood, but it is considered as multifactorial with both genetic and environmental factors involved.

The standardized examination and imaging protocols serve important purpose to diagnose temporomandibular joint (TMJ) arthritis not only to establish an early interventional strategy but also to assess craniofacial growth and the progression of signs and symptoms in those patients. Although the treatment of juvenile idiopathic arthritis (JIA) has changed dramatically over the last decades due to new therapeutic options, TMJ arthritis still can develop during the course of the disease. In clinical experience, TMJs appear to respond less well to the standard of care used to treat other joints. More individualized approach to the patient’s treatment serves as the main goal of personalized medicine. It could be achieved by adopting new methods of medical imaging such as conebeam computer tomography as well as developing reliable biomarkers which may assist with predicting disease type, course, or severity and predicting response to medication.

This article provides an overview of current information on orofacial complications in JIA and its management. Based on information provided in this review, more precise diagnosis, proper tools for recognizing people at risk, and more efficient treatment approaches could be implemented. This may lead to more personalized treatment management strategies of TMJ complications of JIA patients.

## Background

Juvenile idiopathic arthritis (JIA) is the most common childhood rheumatic disease in Europe with an average prevalence of 1 in 1000 children aged 0–15 years. JIA is characterized by the persistent inflammation of at least one joint that lasts for at least 6 weeks. The age at onset of JIA is under 16 years of age [1]. The pathogenesis of JIA is poorly understood, but interaction between environmental and genetic factors has been proposed. The mean annual incidence rate of JIA in Estonia is 21.7 per 100 000 children aged 0–15 years (22.9 in girls and 19.3 in boys) [2].

The prevalence of clinically detectable temporomandibular joint (TMJ) involvement in JIA varies between 38 and 72 %, depending on the diagnostic method used and the JIA subtypes involved [2]. Because joint swelling and other symptoms are seldom reported by patients, TMJ has been described as “the forgotten joint” and diagnostic imaging is considered mandatory to assess JIA involvement [3]. Contrast-enhanced magnetic resonance imaging (MRI) is considered the gold standard to reliably diagnose both acute TMJ arthritis and deformation of the TMJ and may have direct impact on treatment decisions [4]. Biomarkers of diseases are valuable tools for correct diagnosis and for evaluating appropriate treatment protocols. Genetic factors may play a role in determining which individuals are more prone to develop TMJ disorders or in predicting the severity of the disease process. [5] If undetected, TMJ involvement can lead to several functional disabilities, such as reduced mandibular mobility and disorders of the mastication muscles. Orthodontically, the TMJ arthritis may cause significant limitations in sagittal and vertical mandibular growth that can result in severe micrognathia and anterior open bites with significant esthetic and functional restrictions [6]. Early diagnosis of TMJ disorders in JIA patients can lessen individual’s future facial growth problems and dental arch discrepancies. It will also increase the likelihood of treatment success for these patients [7]. It is equally important to monitor craniofacial function and growth through post-diagnostic follow-up clinical examinations. Since the etiology of TMJ pathology is complex, future research faces the challenge of finding methods to effectively differentiate between the etiologies behind JIA-related TMJ arthritis and other subgroups of temporomandibular disorders (TMD) induced TMJ degeneration [8].

Management of TMJ complications in patients with JIA is based on a combination of counseling, pharmacologic therapies, physiotherapy, occlusal appliances, orthodontics, and surgery [9]. Despite the frequency with which the TMJ is involved in patients with JIA, consensus on treatment is lacking. Due to a poor understanding of the etiology or pathogenesis of these diseases and the lack of definitive diagnostic or therapeutic approaches, patients often have to tolerate symptoms, including debilitating pain, that substantially impact their quality of life over extended periods of time [5].

### An overview of JIA and its clinical manifestations

The International League of Associations for Rheumatology (ILAR) has concluded that the term “juvenile idiopathic arthritis” describes seven subtypes of arthritis, which are assessed according to an individual’s clinical features during the first 6 months of disease: oligoarticular JIA, polyarticular JIA (both rheumatoid factor positive and negative), systemic JIA, juvenile psoriatic arthritis, enthesitis-related arthritis, and undifferentiated arthritis [10]. JIA is generally more common in females, although there are differences depending on the subset. The most common JIA subtype is oligoarthritis, which has two forms: persistent (affecting four joints or fewer) and extended (affecting more than four joints) after the first 6 months of the disease [11].

All joints can be affected by JIA, including the temporomandibular. Characteristic joint manifestations of JIA are chronic synovitis, swelling of the joint, arthralgia, and impaired joint mobility. Extra-articular manifestations of the JIA include fever, rash (involvement of internal organs in the case of the systemic subtype), chronic anterior uveitis, and other [12].

Disease onset in smaller children is often abrupt, with the child upon waking unable to walk due to swelling of the joint(s) and morning stiffness a frequent complaint. To alleviate the pain, the joint is held in the position of maximum comfort.

When inflammation occurs within the synovial membrane, an exudative phase occurs (cell infiltration and granulation tissue formation) that leads to an increase in the thickness of the synovial layer. These phases are interrelated with an active phase of the disease, and subsequent immunopathological events perpetuate this initial inflammatory reactions, which result in a chronic condition [13]. With joint tissue destruction, granulation tissue forms and replaces the damaged tissue, forming a pannus. The pannus infiltrates and erodes the articular cartilage and adjacent bone, leading to condylar destruction. As for the TMJ, the condylar growth cartilage resides beneath the superficial articular cartilage layer, so the endochondral mandibular growth can be disturbed when the condyle is being resorbed or eroded [14].

### The uniqueness of the TMJ

The TMJ is different from other joints in the body. The articular surface of TMJ is composed of fibrocartilage, while in the other synovial joints, it is covered by hyaline cartilage. Fibrocartilage is a better material than hyaline cartilage for bearing the large amount of occlusal load placed upon the TMJ [15]. Fibrocartilage is less susceptible to the effects of aging and has a better ability to repair. The cartilage of the mandibular condyle is a secondary cartilage compared to the primary articular cartilage found in all other joints. [16]. This secondary cartilage contains both types of collagen—which may be an adaptation to the complex biomechanical environment of the condylar cartilage—and also a chondroid bone, which allows for highly diverse growth directions and condylar and maxillofacial morphology [17].

Two developmental defects, Hunter-Thompson chondrodysplasia and fibroblast growth factor-receptor 3 (FGFR3) achondroplasia, both of which afflict systemic load-bearing joints but not the TMJ, provide further evidence of the uniqueness of the TMJ [18]. TMJ demonstrates specific organizational-anatomical and developmental differences from all other joints.

From a functional point of view, the two joints—one on each side of the head—are connected by the jawbone. This means that the joints cannot move separately, with one joint influencing the function of the other.

Another factor that makes the TMJ unique is that an individual’s teeth position dictates its function. In order to obtain maximal intercuspation of the teeth, TMJ is forced by the mastification muscles to move so that the teeth fit together properly and this can lead to derangement in the disc-condyle complex.

The articular disc is located between the two articular surfaces of the TMJ. The disc divides the joint into two sections, each with its own synovial membrane. Since the disc is a separate structure and may move independently of the condyle, it can be displaced and cause many problems. This disc-condyle complex derangement may manifest itself in joint noises during jaw movement, pain during functional movements, and limited mouth opening and add further complexity for adequate diagnosis.

The mandibular condylar cartilage is the center of the greatest growth in the craniofacial complex. The growth center is located superficially, unlike in the other synovial joints [19]. The mandibular growth plate lies under a thin layer of fibrocartilage located on the surface of the condylar head [20].

Arthritis in the temporomandibular joint (TMJ) during childhood and youth may affect condylar growth and is considered the main reason for any altered facial structure and changes in the dental occlusion of JIA patients [21].

### Condylar growth impairment of the TMJ in JIA

The reason for this growth impairment is poorly understood, there are several factors that might contribute to this, such as the presence of high levels of proinflammatory cytokines and chronic use of corticosteroids (CS) to control JIA, malnutrition, and immobilization [22]. It has been shown that direct destruction of the condylar region has a high prevalence among patients with JIA—erosive lesions in the condylar region caused by the inflammation process [23]. Body’s growth hormone can be altered by increased proinflammatory cytokines’ levels and prolonged use of medical corticosteroids [13]. When CS is used during the mandibular growth phase, a premature closing of the epiphyseal bone may occur [24]. These alterations seem to become more evident in patients aged 9–12 years. During this period is a growth spurt which is accompanied by faster jaw growth [25].

With JIA, this growth disturbance has been associated with a number of clinically significant outcomes, including decreased chewing ability, malocclusion, and micrognathia [26].

### Craniofacial growth and dentoalveolar development in JIA patients

The destructive changes in the mandibular condyle seem to lead to a change in mandibular position, with a more anterior position of the condyle in the glenoid fossa, and to its reduced vertical growth. Both the condyle–fossa positional change and reduced vertical condylar growth lead to a posterior rotation of the mandible, mandibular retrognathia, and malocclusion [27].

The condyle can grow until the third decade of life [28], which is different from the suture of the maxilla that is almost completed at 10 years of age [29]. If condylar growth is inhibited, muscles of mastication force the lower jaw to rotate backwards in relation to the cranial base, giving a steeper mandibular plane angle and a shorter posterior facial height. The result is a more convex profile and reduced mandibular protrusion [30]. If only one TMJ is affected by JIA, facial asymmetry occurs; as in this case, only one area of mandibular growth is compromised, while the other shows normal growth [13].

Posterior rotation of the mandible and the subsequent decrease in mandibular dimensions in patients with JIA can lead to an anterior open bite [31]. This open bite can result in mastication overload of the posterior teeth, muscles, and joint structures, which may cause muscle imbalances and intracapsular disorders.

From an orthodontic perspective, JIA patients show a significantly greater prevalence of anterior open bite, an increased orthodontic treatment need according to the morphological component of the Index of Orthodontic Treatment Need (IOTN) and less normal bite than healthy children [31]. They have a steeper occlusal plane, tilting of the lower incisors, and a reduced height of the ascending ramus [32].

Various JIA subtypes can impact orofacial structures with different amount. The greatest alterations are noticeable with the polyarticular subtype compared to other JIA subtypes [27]. A mandibular growth disturbance is also dependable on child’s age at disease onset. Longer exposure to treatment or to the harmful effects of the disease will increase the likelihood for interfered development of the mandible. [33].

The teeth and gingiva can be indirectly affected by JIA owing to physical limitations of the superior limbs of patients, which make performing adequate oral hygiene difficult and contributes to a higher incidence of dental and gingival problems among JIA patients when compared to healthy children [25]. In a small study of 16 JIA patients with a mean age of 9.3 years, the mean salivary concentrations of calcium, phosphorus, potassium, lysozyme, and IgA were significantly lower in the JIA patients when compared to healthy children [34].

Children with JIA also have a higher prevalence of headaches, neck pain, and jaw dysfunction than non-JIA controls, both at the time of TMJ disease diagnosis and at the long-term follow-up [35].

### The diagnosis of TMJ arthritis

A correct diagnosis is important to distinguish between dental malocclusion and bone discrepancy, thus differentiating the normal bone growth trajectory from an abnormal growth caused by a pathological condition [13]. Diagnosis of TMJ arthritis includes a questionnaire for history and epidemiological background, a pain anamnesis, and an examination that consists of an analysis of the TMJ, the masticatory muscles, and the interaction of TMJ function with the articular disc [8].

As the symptoms of TMJ arthritis may also manifest in its surrounding structures, special care must be paid to the whole orofacial complex during clinical examination. Symptoms are more manifested during mouth opening and chewing and are most commonly noticeable in the TMJ area and the masseter muscle region [8]. As with other joints, morning stiffness of the TMJ is also a frequent complaint. TMJ symptoms and a physical examination are not reliable methods for the detection of TMJ arthritis. Many initially asymptomatic cases during clinical examination are later diagnosed to have TMJ damage by radiograhic imaging methods [35]. Pain sensation among JIA patients varies considerably, but usually, pain is moderate and fluctuates over time [36].

Like with other chronic diseases, TMJ arthritis-related orofacial symptoms often fluctuate and can be difficult to recall during the clinical examination. It is recommendable to introduce a standardized patient questionnaire in which the patients report their orofacial symptoms within the last 2–4 weeks [8].

Reduced mandibular range of motion and asymmetric mouth opening are the two most common clinical signs associated with TMJ involvement [37]. Maximal mouth-opening capacity (MOC) increases linearly with age but shows a wide range among children of the same age group. Mean MOC (range) is 45 mm (25–69) for girls and 45 mm (25–70) for boys [38]. A MOC of ≤29.5 mm can be considered limited in children up to 3 years old; a MOC of 34.5 mm and of 39.5 mm is limited for children up to 6 years old and for those older than 7 years, respectively [39]. Asymmetry in relation to the facial vertical midline during opening of the mandibula may be a consequence of the shortened condyle on the side of deviation but may also indicate an intercapsular disorder on that side.

There are no standardized protocols to determine intervals between routine clinical examinations for JIA patients, and this should be adapted to each individual patient regarding disease activity, age, complaints, and detectable TMJ involvement. The current age of the child and related future craniofacial growth potential should also be a decisive factor in determining the interval. Disease onset before or during the pubertal growth spurt can have a larger impact on craniofacial development, and such patients should be recalled more often [8].

There are no specific validated examination protocols for patients with JIA. An expert-based classification and diagnostic system was proposed in 2014 called Diagnostic Criteria for TMD (DC/TMD) [40]. Being regarded as a gold standard for TMD diagnosis, this criterion consists of pain anamnesis, a questionnaire for a case history, and a complex physical examination of TMJ and masticatory muscles. It is considered a valid screener and diagnostic criteria for detecting and differentiating any pain-related TMDs [40]. Using this protocol for JIA patients is limited because it does not evaluate granofacial growth or morphology [8]. A task force within the EuroTMJoint research network is preparing recommendations for a standardized clinical orofacial examination of patients with JIA, which are expected to be published in 2016 on the webpage of the euroTMJoint network [8].

Diagnostic imaging is considered mandatory when assessing JIA involvement in TMJ [41]. Panoramic radiography (OPTG) has the advantage of being available and simple and is still used in many studies. Magnetic resonance imaging (MRI) has become the new standard for examining TMJ, as it allows noninvasive evaluation of the joint.

With X-rays, the most commonly described feature of TMJ is flattening of the condyle, which is usually a sign of remodeling of the condyle [42]. The temporal bone may also be involved in JIA, but it cannot be evaluated using panoramic radiography. TMJ function is often impaired in JIA patients. In children with JIA, maximal condylar translation was less than one half of that observed in the healthy controls, which was noticeable even when JIA patients had normal mouth-opening capacity [43].

There are some imaging findings that correlate to clinical signs and symptoms, which may help with establishing an early diagnosis of TMJ arthritis. Clinical assessment of limited mouth-opening capacity, mandibular deviation during mouth opening, and reduced condylar translation is strongly associated with TMJ involvement on imaging in a number of studies [43, 44]. Clinical predictors of TMJ involvement in studies using conventional radiographic methods and MRI are young age at onset (<4 years), long disease duration, systemic or polyarticular disease subtype, and extended disease course [26, 45, 46]. Many clinicians still use X-ray for imaging during early phases of TMJ arthritis. However, during early phases, OPTG has poor sensitivity, and it appears to be better suited for studying TMJ bony changes later in the disease course [45].

Panoramic examination is simple and accessible diagnostic method at most clinics and should be used as a foundation to get an overview of the TMJ before more sophisticated imaging methods are performed.

With the use of MRI, the condition of all joint components can be assessed and inflammatory activity detected. Long-standing TMJ involvement has been shown to cause bone deformation, frequently with secondary osteoarthritis and quite often with mild contrast enhancement [42]. As shown in the subsequent MRI examination, clinical symptoms were found to be poor predictors of the presence or absence of TMJ involvement in JIA patients [46, 47]. Active TMJ involvement on MRI was associated with higher incidence of recent involvement of other joints which signifies the need for early diagnosis of TMJ involvement to help in directing clinical management and obtain early intervention [48].

An early diagnosis of TMJ arthritis is essential to prevent condylar destruction. At the time when clinical and morphological signs like retrognathism or jaw asymmetry become obvious, the condyles are already irreversibly damaged [49].

TMJ arthritis and TMJ involvement were significantly associated with a younger age at onset of JIA [50]. Early TMJ MRI led to changes in the treatment in 62 % of patients with 60 patients getting additional joint injections and 9 patients getting systemic medication. Therefore, it is recommended to perform TMJ MRI in young children even if they require sedation, as they have an increased rate of TMJ involvement which can stay undetected during clinical examinations [4].

Ultrasound is routinely used to detect synovitis in seve62 % of patients with 60 ral joints and has been shown to be useful for monitoring the activity and course of the disease process in children with JIA but has low sensitivity compared to MRI [51].

The introduction of the conebeam computer tomography (CBCT) system specifically designed for use in dentistry provides a definite advantage over other techniques due to its low radiation dose to the patient, lesser cost for equipment, and ability to provide accurate 3D images of the surface of the condyle. Diagnostic information obtained is limited to the osseous joint components and is not suitable for soft tissue evaluating soft tissues [52].

CBCT imaging technology has been used for an image-guided access to the superior joint space and for arthroscopic examination and treatment of individuals with disc perforations or adhesions [5].

### The genetics of juvenile idiopathic arthritis

As with most complex diseases, such as JIA, different genomic regions are suggested to contribute to the overall disease risk of JIA [53]. Genetic factors may play a role in determining which individuals are more prone to develop TMJ disorders or in predicting the severity of the disease process [5]. Evidence for a genetic component regarding the pathogenesis of JIA has come from both twin and family studies: siblings of those affected by JIA have a 15- to 30-fold increase in the prevalence of JIA. A concordance rate of 25 % for JIA was found in monozygotic twins [54]. JIA represents a clinically heterogeneous group of conditions, and given the relative rarity of the disease, there are varied strategies associated with studies of the genetic background of JIA.

It has been suggested that several genetic factors are shared with other autoimmune diseases, which are supported by the candidate gene studies [55].

Studies have shown that polymorphisms in the serotonin transporter gene [56] and adrenergic receptor beta gene 2 [57] cause an increase in the likelihood of developing TMJ disorders. Also, polymorphism in the estrogen receptor gene causes an increase in pain susceptibility in patients with TMJ osteoarthritis [58]. The differences in findings between studies on the heritability of TMJ disorders are likely resulted from the inclusion of the many different subtypes of the diseases with some having a greater genetic component while others have minimal or no genetic basis [5].

In the last few years, genome-wide association studies (GWAS) have been shown to be highly effective in discovering new susceptibility loci for common complex traits but have not contributed much in the field of JIA research [59].

Strong evidence supports HLA class I and II alleles as having an important role in the pathogenesis of different subtypes of JIA [60]. Several studies have shown significant correlations between the antigens associated with early-onset oligoarticular, RF-negative polyarticular, and RF-positive polyarticular JIA [60]. There are also a number of non-HLA genes that have been tested for the association with JIA, most of them belonging to the immunity-related genes [59]. The genetic background of TMJ arthritis has not yet been specifically addressed. Advances in understanding the genes that contribute or predispose to TMJ disorders may make it possible to identify patients who are at risk for developing TMJ disorders and enable the implementation of strategies to prevent the disease.

### Biomarkers for juvenile idiopathic arthritis

Juvenile idiopathic arthritis consists of a heterogeneous group of disorders. Clear distinctions between the various subtypes are difficult because of unknown etiology and complex genetic component. The use of reliable biomarkers would be beneficial for diagnostic purposes or for monitoring disease course. A biomarker is a small component which is easily measurable in accessible patient material, e.g., blood, urine, or saliva, and is ideally obtained using a relatively noninvasive approach [61]. Biomarkers may be helpful with predicting disease type, course, or severity. They may also help to predict response to medication and identify those children who have obtained clinical remission [61]. Several biomarkers are already in widespread use in routine care of JIA such as antinuclear antibody (ANA), C-reactive protein (CRP), and rheumatoid factor (RF). The presence of positive serum antinuclear antibody (ANA) has been revealed in several studies to be associated with an increased risk of chronic anterior uveitis in JIA [62]. Two polyarticular forms of JIA are distinguished by the absence or presence of serum autoantibody, known as rheumatoid factor (RF). These two clinical subgroups of JIA are distinct in their genetics, age of onset, and prognosis [63]. In extended oligoarthritis, five or more joints are affected after the first 6 months; joint’s inflammation might be highly erosive and may be treatment refractory. It has been shown that compared to persistent oligoarticular JIA, CD4:CD8 ratio in synovial fluid is lower and the levels of the chemokine CCL5 are higher in extended-to-be oligoarticular JIA [64].

The proinflammatory S100 proteins, S100A8/9 (also known as calprotectin or myeloid-related protein (MRP) 8/14), and S100A12 have been described to be sensitive markers for disease activity in JIA, and both correlate well with physicians’ assessment of disease or with active joint inflammation [65]. The level of S100 proteins in serum may also show whether a patient will respond to initial treatment with methotrexate [66]. In another approach, evaluating gene expression profiles in children with JIA before and after MTX showed three SNPs in the SLC16A7 gene that may contribute to genetic variability in MTX response in [67].

Biomarkers could also be valuable to identifying patients at risk of disease relapse after discontinuation of MTX treatment helping to detect a presence of subclinical disease activity [61].

Many biomarkers are developed for the adult population and are later applied to children.

However, some diseases only occur in children, and the pathogenesis of disease is often different in children compared with adults [65]. For example, MTX is a folate antagonist and it is essential for numerous bodily functions. Children are receiving higher absolute doses of MTX for clinical efficacy which might be related to serum folate concentration decrease with age.

Measurement of the polyglutamated form of the drug in erythrocytes has been shown to correlate with improved outcomes in adult rheumatoid arthritis patients [68] but not in children with JIA [69]. The different antifolate effect of MTX might be related to changes in folate concentration during growth.

Validated pediatric biomarkers are vital in caring for JIA patient and should be objective of modern management of arthritis. To obtain such goal, multi-center and international collaborations are needed to support biomarker verification or validation in large multicenter studies [61].

### Treatment of JIA

The main objective of the treatment of JIA is to reduce an individual’s pain and prevent or control joint damage and loss of function [70]. JIA is a chronic disease that is characterized by periods of remission and flare-up. Early aggressive treatment is targeting longer or permanent remission and is aimed at preventing body’s own immune system to destroy joint’s tissues. Pharmacologic management consists of nonsteroidal anti-inflammatory drugs (NSAIDs), disease-modifying antirheumatic drugs (DMARDs), biologic agents, and intra-articular and oral steroids [71]. NSAIDs ease pain and reduce inflammation but do not prevent joint damage. Intra-articular corticosteroids offer a very rapid relief of symptoms and can spare the need for systemic therapy among patients with persistent oligoarticular arthritis. Intra-articular corticosteroids might be beneficial in cases where active TMJ arthritis persists despite a use of systemic therapy, and other joints remain in remission [72].

Methotrexate remains the most widely used conventional DMARD in the management of JIA. It has long-term beneficial effects in controlling disease activity, and it reduces the exposure to other medications such as prednisolone and NSAIDs. However, methotrexate can be associated with a number of potentially serious adverse effects, and therefore, its use requires careful monitoring using blood tests and clinical supervision from a medical specialist [73].

The development of biologics has changed the treatment of JIA, with a variety of new drugs that target key cytokines and other inflammatory mediators. They are co-administered with methotrexate. Multiple categories of biologics exist, targeting different key cytokines underlying the inflammatory response with different disease subtypes. Most common biologics are aimed at targeting tumor necrosis factor (TNF), in case of systemic disease, interleukin (IL)-1 and IL-6. There are also drugs that aim at T cell blockade and B cell depletion [74]. Better understandig of the etiopathogenesis of the subcategories of JIA makes it easier to choose adequate treatment to the right child. Current consensus is that most children with non-systemic JIA respond well to TNFi and MTX and that most children with sJIA respond well to IL-1 and IL-6 blockade [75].

Although the systemic treatment of JIA has improved significantly, TMJ arthritis still can develop during the course of the disease, even if patients are treated with biologics [76].

The management of TMJ pathology in patients with JIA is based on a combination of interventions, such as counseling, pharmacologic therapies, physiotherapy, occlusal appliances, orthodontics, and surgery [77]. The main treatment goals for these patients are to improve esthetics and function, reduce pain, and avoid progression of the process.

It is advisable to educate the patient about the possibilities for reducing the load put on the joint. Patient education should begin by informing the patient that the teeth should only be in contact during chewing, speaking, and swallowing. At all other times, the jaw should be positioned with the teeth apart. If the patient has an intracapsular disorder, it may be advisable to chew with the same side, because this increases interarticular pressure in the contralateral joint and decreases intra-articular pressure in the ipsilateral joint [78]. Warm heat may be helpful with chronic pain. Any activities that increase pain should be resisted.

Patients who experience TMD symptoms often decrease the use of their jaw because of pain. If disuse is prolonged, the muscles can become shortened and atrophied. The patient should therefore be instructed in self-administering exercises that largely fall into four types: passive muscle stretching, assisted muscle stretching, resistance exercises, and postural training [79]. These exercises are repeated ten times, six sessions a day. If any of them elicit pain, they are discontinued. The main objective of these exercises is to help restore normal function and range of movement.

When nocturnal clenching or bruxism is suspected or there exists an occlusal instability, the use of a stabilization appliance is indicated to decrease muscle hyperactivity and correct any occlusal discrepancies. Because the etiology of temporomandibular symptoms are often complex, it is advisable that any initial therapy should generally be reversible and noninvasive. When an appliance helps to modify an etiologic factor, it might also be considered as a diagnostic tool.

The most common type of appliance is a stabilization appliance. It provides an occlusal relationship considered optimal for the patient. When it is in place, the condyles are in their most musculoskeletally stable position at the time the teeth are contacting evenly and simultaneously. Canine disocclusion of the posterior teeth during eccentric movement is also provided. The treatment goal of the stabilization appliance is to eliminate any orthopedic instability between the occlusal position and the joint position, thus removing this instability as a causative factor of TMD [78].

There is evidence that intra-articular corticosteroid therapy (IACS) is effective in many patients with TMJ arthritis, and although its short-term safety profile has been well established, there remain concerns about long-term effects on mandibular growth [80].

Orthodontic treatment is carried out during the remission phase of JIA and follows the protocol for non-JIA children. Early treatment should be aimed at supporting the symmetric growth, avoiding compensations, and providing space for the normal development of the dentoalveolar area [81].

Avoidance of mandibular and craniofacial growth disturbances is a primary aim in TMJ arthritis management in children since the magnitude of the treatment involves substantial and clinically challenging orthopedic and possibly surgical interventions that proceed over a long period which requires excellent patient cooperation [82].

### Conclusions

The etiological factors of JIA are largely unknown. Early and correct TMD diagnosis in patients with JIA is important in order to treat and prevent a negative effect of the chronic inflammatory disease process primarily on the TMJs and secondarily on craniofacial development. Etiology and pathogenesis of JIA is poorly understood and lack of person tailored therapeutic approaches impact patients’ quality of life through their lives. The screening for TMJ arthritis, description of possible dentofacial growth disturbances and malocclusions, and registering the Index of Orthodontic Treatment Need should be beneficial to outline a long-term treatment plan for JIA patients. Magnetic resonance imaging is the most recommendable examination method but requires sedation in young children. The introduction of the conebeam computer tomography has opened up new opportunities for deriving additional diagnostic information; it is a noninvasive and repeatable tool for individualization of treatment. Future classification of JIA might be revisited when clinical and laboratory features are accompanied by better understanding of genetic predispositions for gene expression and cytokine profiles. Genetic analysis, novel biomarkers, and imaging methods will improve the specificity of diagnostic and therapeutic approaches for diseases of the TMJ [5].

Multi-center and international collaborations are needed to gain insights into how genetics and environment are intervened in the onset and disease course of JIA and in their role leading to the development of TMJ arthritis.

To achieve these targets, propagation of multidisciplinary expertise in dentistry and networking of scientific and dental/medical centers is mandatory, in order to shift the paradigm from reactive medical services to predictive, preventive, and personalized medicine in dentistry [45].

## Abbreviations

ANA: antinuclear antibody
CBCT: conebeam computer tomography
CS: corticosteroids
DMARD: disease-modifying antirheumatic drug
FGFR3: fibroblast growth factor-receptor 3
GWAS: genome-wide association study
IOTN: index of orthodontic treatment need
JIA: juvenile idiopathic arthritis
MOC: mouth-opening capacity
MRI: magnetic resonance investigation
NSAID: nonsteroidal anti-inflammatory drug
OPTG: orthopantomogram
RF: rheumatoid factor
SNP: single nucleotide polymorphism
TMD: temporomandibular dysfunction
TMJ: temporomandibular joint
